# Identification of New Chemoresistance-Associated Genes in Triple-Negative Breast Cancer by Single-Cell Transcriptomic Analysis

**DOI:** 10.3390/ijms25136853

**Published:** 2024-06-22

**Authors:** Spyros Foutadakis, Dimitrios Kordias, Giannis Vatsellas, Angeliki Magklara

**Affiliations:** 1 Biomedical Research Foundation, Academy of Athens, 11527 Athens, Greece; gvatsellas@bioacademy.gr; 2 Biomedical Research Institute-Foundation for Research and Technology, 45110 Ioannina, Greece; d.kordias@hotmail.com; 3 Department of Clinical Chemistry, Faculty of Medicine, School of Health Sciences, University of Ioannina, 45110 Ioannina, Greece; 4 Institute of Biosciences, University Research Center of Ioannina (URCI), 45110 Ioannina, Greece

**Keywords:** single-cell RNA-sequencing, bulk RNA-sequencing, triple-negative breast cancer, chemoresistance, transcriptomics, 3D spheroids

## Abstract

Triple-negative breast cancer (TNBC) is a particularly aggressive mammary neoplasia with a high fatality rate, mainly because of the development of resistance to administered chemotherapy, the standard treatment for this disease. In this study, we employ both bulk RNA-sequencing and single-cell RNA-sequencing (scRNA-seq) to investigate the transcriptional landscape of TNBC cells cultured in two-dimensional monolayers or three-dimensional spheroids, before and after developing resistance to the chemotherapeutic agents paclitaxel and doxorubicin. Our findings reveal significant transcriptional heterogeneity within the TNBC cell populations, with the scRNA-seq identifying rare subsets of cells that express resistance-associated genes not detected by the bulk RNA-seq. Furthermore, we observe a partial shift towards a highly mesenchymal phenotype in chemoresistant cells, suggesting the epithelial-to-mesenchymal transition (EMT) as a prevalent mechanism of resistance in subgroups of these cells. These insights highlight potential therapeutic targets, such as the PDGF signaling pathway mediating EMT, which could be exploited in this setting. Our study underscores the importance of single-cell approaches in understanding tumor heterogeneity and developing more effective, personalized treatment strategies to overcome chemoresistance in TNBC.

## 1. Introduction

Triple-negative breast cancer (TNBC) is characterized by the absence of estrogen (ER) and progesterone (PR) receptors and a lack of overexpression of the human epidermal growth factor receptor 2 (HER2). It is considered to be the most aggressive type of mammary tumor, with high rates of recurrence within the first 3–5 years after diagnosis, a tendency for distant, incurable metastases, and a lower overall survival compared to other breast cancer subtypes [1]. As a result, even though TNBC makes up only 15–20% of all breast cancer diagnoses, it accounts for ~25% of breast cancer-associated fatalities [2]. These grim characteristics are largely due to the limited treatment choices for this disease. The lack of expression of the three biomarkers (ER, PR, and HER2) renders the associated therapies unsuitable for the successful management of TNBC. Furthermore, the development of other targeted treatments has been challenging due to its high inter-tumoral heterogeneity and the absence of recurrent, treatable driver mutations [3]. Consequently, systemic chemotherapy, including taxane- and anthracycline-based schemes, remains the mainstay treatment option for most TNBC patients, with initially good response rates [1,3,4]. Nevertheless, a large fraction of patients present with tumor recurrence, often leading to distant metastasis and death [5], suggesting that either they develop resistance during treatment (adaptive or acquired chemoresistance) and/or are inherently less responsive (innate chemoresistance). Therefore, combatting chemoresistance in TNBC is a clinically urgent need that may be addressed by untangling the molecular underpinnings of the disease, so that more effective, personalized treatment strategies can be developed.

Genomic and transcriptomic profiling have considerably improved our understanding of tumor biology, opening up new therapeutic avenues for many types of cancer. However, the overwhelming majority of transcriptomic studies include only bulk analyses of cancer models and human tumor samples, failing to capture intratumoral heterogeneity and overlooking the gene expression statuses of minor subpopulations. This is particularly problematic when investigating the mechanisms of chemoresistance in TNBC, where first-line therapies eliminate the bulk of cancer cells and tumor relapse is probably due to a few remaining cells that already harbor advantageous properties for evading cytotoxicity. To overcome this limitation, transcriptome profiling at the single-cell level has recently emerged as a powerful tool, providing valuable insights into critical disease processes, including the development of drug resistance [6]. In contrast to bulk RNA-sequencing (RNA-seq), which averages gene expression across a multitude of cells, single-cell RNA-seq (scRNA-seq) analyzes the transcriptome of each cell individually, enabling the identification of transcriptionally distinct cell populations within tumors, including rare ones that may drive chemoresistance.

Despite the undisputed value of two-dimensional (2D) cultures of cancer cell lines in advancing the field of cancer biology, these in vitro models fail to reconstitute the complexity of human tumors, mostly due to a lack of interactions with the components of the tumor microenvironment (TME). Scaffold-based three-dimensional (3D) cell culture models, also known as spheroids, are spherical multicellular entities, usually generated from cell lines, that show a low complexity in histological organization, but recapitulate the gradients of oxygen and nutrients, as well as the cell–cell and cell–extracellular matrix (ECM) interactions, of the in vivo tumor [7].

In this study, we employed both bulk RNA- and scRNA-seq to investigate the transcriptional status of TNBC cells, grown in 2D or 3D cultures, prior to and after the development of resistance to two widely used chemotherapeutic drugs, paclitaxel and doxorubicin. Our main goal was to delineate the heterogeneity in these TNBC models and uncover the gene expression programs that support drug resistance in minor groups of chemoresistant populations that go undetected by bulk RNA-seq. Analysis of the data from the two transcriptomic techniques yielded similar results to a large degree, yet several low-abundance gene expression changes in the chemoresistant cells were detected only by scRNA-seq. We hypothesize that these genes may constitute novel therapeutic vulnerabilities that can be targeted by combinatory treatment schemes, including repurposed drugs. We also found that some markers, strongly upregulated in the majority of chemoresistant cells, were already expressed in rare pools of cells before treatment, suggesting that less drug-sensitive subpopulations preexist and are selected upon drug exposure. Our results from the two different culture models used (2D and 3D) largely overlapped, however, they also presented some discrepancies that are discussed in the text.

Overall, the findings of our study underscore the complexity of chemoresistance in TNBC and highlight the utility of scRNA-seq in unraveling the transcriptional heterogeneity underlying this phenomenon.

## 2. Results

### 2.1. Single-Cell Transcriptome Analysis of SUM159 TNBC Cells Indicates a Partial Shift to a Highly Mesenchymal Phenotype upon Acquisition of Chemoresistance

Seeking to unravel the mechanisms of acquired chemoresistance in TNBC at the single-cell level and identify new potential therapeutic targets, we performed scRNA-seq experiments on SUM159 cells prior to and upon the establishment of resistance to paclitaxel or doxorubicin. The SUM159 paclitaxel-resistant cells had been previously described [8] and the SUM159 doxorubicin-resistant cells were newly generated.

The scRNA-seq experiments were performed using a commercially available panel that contained primer pairs targeting 389 breast-cancer-associated genes (see Section 4). First, we sought to leverage the power of this technique to examine the transcriptional heterogeneity within each cell population. We collected data from 18,233 TNBC cells grown in a monolayer across all conditions, and after quality control, batch effects’ removal, and a principal component analysis with Scanpy [9], a total of 12,082 single cells were visualized using uniform manifold approximation (UMAP) plots. The Louvain clustering algorithm was used to identify transcriptional cell subpopulations based on cluster-specific expression patterns, and the 100 most variable genes were obtained using a t-test statistic after regressing out the effect of cell cycle genes (see Section 4).

**Figure 1:**
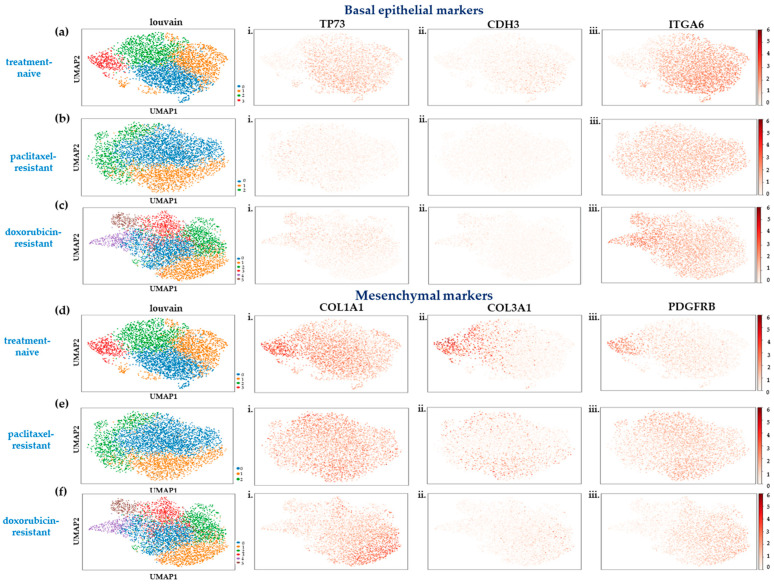
In the upper panel, UMAP plots of the SUM-159 (**a**) treatment-naïve, (**b**) paclitaxel-resistant, and (**c**) doxorubicin-resistant cells grown in monolayer are shown, as well as the individual single-cell expression patterns of the basal epithelial marker genes (**i**) *TP73*, (**ii**) *CDH3*, and (**iii**) *ITGA6* in these cell states. In the lower panel, (**d**–**f**) the same UMAP plots (**d**–**f**) are shown along with the individual single-cell expression patters of the mesenchymal marker genes (**i**) *COL1A1*, (**ii**) *COL3A1*, and (**iii**) *PDGFRB*.

It is known that a population of SUM159 cells represents a continuum, ranging from an epithelial-like cell state with stemness properties to a highly mesenchymal one [10]. To determine which cell state dominated each cluster in the single-cell datasets, we re-analyzed publicly available bulk RNA-seq data generated from derivatives of this cell line that represented epithelial-like and highly mesenchymal populations [10] to generate gene signatures for each cell state. We intersected these with our own data to characterize the cell clusters identified in each cell line, and the results are presented in Figure 1.

In the treatment-naïve SUM159 cells, four clusters were identified (Figure 1a,d). Cluster 3 (9% of the cell population, depicted in red in Figure 1a,d) represented a highly mesenchymal state, and was characterized by a very weak expression of the epithelial markers *TP73*, *CDH3*, and *ITGA6* (Figure 1(ai–iii)) and upregulation of the mesenchymal genes *COL1A1*, *COL3A1*, and *PDGFRB* (Figure 1(di–iii)) compared to the other clusters. On the other hand, the most populous cell group, cluster 0, encompassed 37% of the cells (depicted in blue in Figure 1a,d) and expressed higher levels of the epithelial markers (Figure 1(ai–iii)) and lower levels of the mesenchymal markers (Figure 1(di–iii)), probably representing an epithelial-like state. Clusters 1 and 2 (depicted in orange and green respectively, in Figure 1a,d) could not be assigned to either cell phenotype and possibly represented intermediate states in the epithelial-to-mesenchymal spectrum.

Analysis of the scRNA-seq data in the paclitaxel-resistant cells showed a marked, almost universal, loss of expression of the epithelial markers *TP73* and *CDH3* (Figure 1(bi,ii), respectively), and uncovered three subpopulations with different gene expression patterns (Figure 1b,e). Cluster 1 was assigned to the highly mesenchymal state (28.6% of the population, depicted in orange in Figure 1b,e) due to the overexpression of markers *COL3A1* and *PDGFRB* (Figure 1(eii,iii)). In contrast, cluster 2 (18.8% of the population, depicted in green in Figure 1b,e) exhibited relatively higher *TP73* (Figure 1(bi)) and lower mesenchymal gene levels (Figure 1(ei–iii)) than the other clusters, representing a more epithelial-like cell subpopulation. The cells in cluster 0 (depicted in blue in Figure 1b,e) may represent a transition state or a more heterogeneous population with both epithelial and mesenchymal features.

Interestingly, the development of adaptive resistance to doxorubicin favored an increase in transcriptional heterogeneity, as six cell clusters showed (Figure 1c,f). The gene signature related to a highly mesenchymal state was evident in cluster 1 (24.3% of the population, depicted in orange in Figure 1c,f), with cells expressing lower levels of epithelial markers (Figure 1(ci–iii)) and higher levels of mesenchymal ones (Figure 1(fi–iii)). On the other hand, clusters 4 (6.9% of the population, depicted in purple in Figure 1c,f) and 5 (4.9% of the population, depicted in brown in Figure 1c,f) seemed to be situated at the epithelial end of the EMT spectrum, exhibiting an overexpression of the epithelial genes (Figure 1(ci–iii)) and a downregulation of the mesenchymal ones (Figure 1(fi–iii)). Clusters 0, 2, and 3 (depicted in blue, green, and red, respectively, in Figure 1c,f) were assumed to represent hybrid E/M cell states.

These data suggest that a shift occurs to a highly mesenchymal state when TNBC cells adapt to drug exposure. This shift, though, is only partial, with a moderate increase (~3-fold) in the number of cells displaying this phenotype after the development of chemoresistance. Most cells, both treatment-naïve and chemoresistant, seemed to reside in a range of hybrid states across the EMT spectrum, depicting variable transcriptomic profiles.

### 2.2. Differential Gene Expression Analysis of the scRNA-Seq Data Reveals New Resistance-Associated Genes in Chemoresistant TNBC

To identify the genes mediating the development of acquired chemoresistance in TNBC, we performed a differential expression analysis of the scRNA-seq data between the treatment-naïve and each of the drug-resistant cell populations.

**Figure 2:**
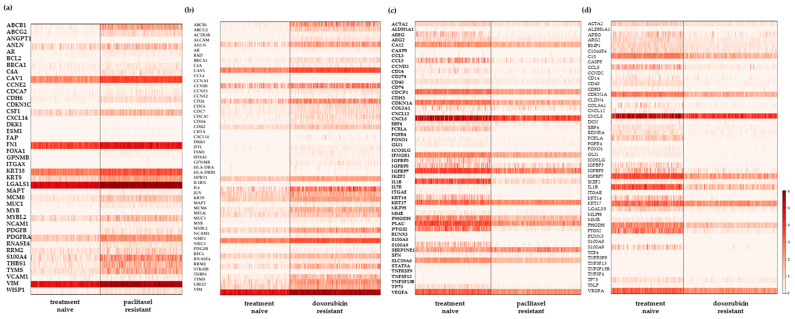
Heatmaps showing the differentially expressed genes in the SUM159 chemoresistant compared to the treatment-naive cells grown in monolayer. (**a**,**b**) Show the upregulated genes and (**c**,**d**) show the downregulated genes in paclitaxel- and doxorubicin-resistant cells, respectively. The *PTPRC* gene is missing from (**a**) and the *CD244* and *TCL1A* genes are missing from (**b**) due to their very low counts in the treatment-naïve cells that excluded them from the filtered count matrix.

In the 2D-grown cells, a pseudobulk analysis of counts of the scRNA-seq data revealed 42 and 55 upregulated genes in the paclitaxel- (Figure 2a) and doxorubicin- (Figure 2b) resistant cells, respectively. We compared these results with our previously published bulk RNA-seq data for the paclitaxel-resistant cells [8], while we performed new transcriptomic experiments for the doxorubicin-resistant ones. We identified 20 common genes in the first case (Figure S1a) and 25 in the latter (Figure S1b). As expected, most of these genes were expressed both in higher levels and in a higher fraction of the population in the chemoresistant cells compared to the treatment-naïve ones (Figures S1a,b). Regarding downregulated genes, the analysis of the scRNA-seq data identified 52 and 50 such genes in the paclitaxel- (Figure 2c) and doxorubicin- (Figure 2d) resistant cells, respectively. Comparison with the corresponding bulk RNA-seq data revealed 18 and 15 common genes, respectively, as shown in Figures S1c,d.

**Figure 3:**
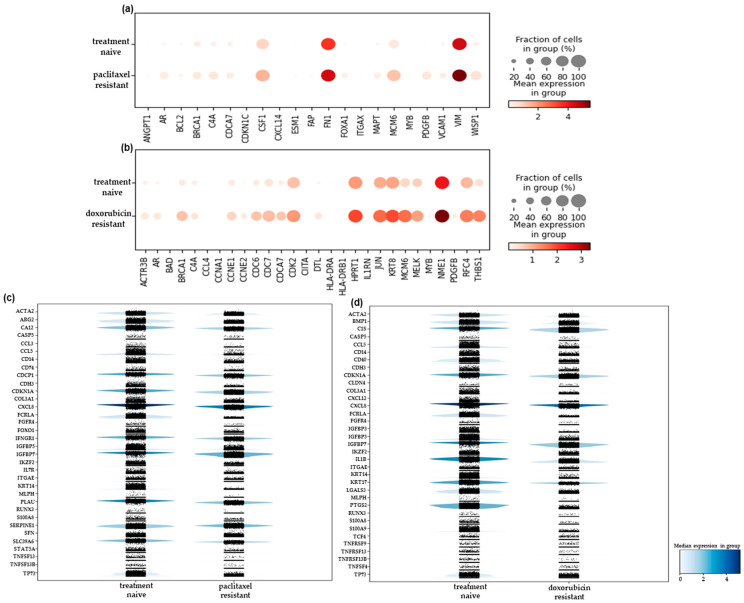
Differentially expressed genes in the 2D-cultured chemoresistant SUM159 cells compared to the treatment-naïve ones that were identified only by single-cell (sc) RNA- and not by bulk RNA-sequencing. Dotplots depicting genes that are upregulated in the (**a**) paclitaxel- and (**b**) doxorubicin-resistant cells, respectively. Stacked violin plots depicting genes that are downregulated in the (**c**) paclitaxel- and (**d**) doxorubicin- resistant cells, respectively. Each dot represents the normalized gene expression per cell. The *PTPRC* gene is missing from (**a**) and the *CD244* and *TCL1A* genes are missing from (**b**) due to their very low counts in the treatment-naïve cells that excludes them from the filtered count matrix.

The targeted nature of the scRNA-seq experiments afforded a higher sensitivity to detecting lowly expressed transcripts that were not identified by the bulk RNA-seq experiments, sequenced at roughly the same depth (around 5–10 million reads). Thus, we focused on the upregulated genes in the drug-resistant cells that were uniquely identified by scRNA-seq, suggesting that they were overexpressed only by a very small percentage of cells. In the paclitaxel-resistant cells (Figure 3a), the most highly upregulated genes included ones that have been associated with breast cancer metastasis, such as the platelet-derived growth factor *PDGFB* [11] and the *MYB* oncogene [12]. Interestingly, *MYB* has also been implicated in chemoresistance in colorectal [13] and ovarian [14] carcinomas, as well as in endocrine resistance in luminal breast cancer cells [15]. Another upregulated gene, *FOXA1,* a multi-faceted transcriptional regulator, is an established marker of a good prognosis in luminal breast cancer [16]. Even though it is considered to be a “luminal” gene, there are several reports of its overexpression in TNBC, where it is mostly associated with a poor prognosis and short overall survival [17,18,19]. In addition, the ectopic overexpression of *FOXA1* in TNBC MDAMB231 cells led to increased resistance to paclitaxel and doxorubicin [20]. These findings are in accordance with our own data, suggesting that *FOXA1* may be a marker of chemoresistance in this subtype of breast cancer. It is noteworthy that all three genes were upregulated in very small fractions of the resistant cells (Figure 3a), making their detection by bulk RNA-seq unattainable at a conventional sequencing depth. *MYB* and *PDGFB* were also present among the highest upregulated genes in the doxorubicin-resistant cells (Figure 3b), supporting the hypothesis that they play important roles in the development of chemoresistance in TNBC. *CCNE2*, which encodes for cyclin E2, was also upregulated in these cells (Figure 3b). An elevated expression of this gene has been associated with tamoxifen resistance in ER^+^ breast cancer [21], and it was recently implicated in doxorubicin resistance in MDAMB231 cells [22].

The downregulated genes in the resistant cells that were uniquely identified by scRNA-seq are presented in Figure 3c,d. Reduced expression of the IGFBP5 and IGFBP7 members of the insulin growth factor-binding proteins (IGFBP) was a common feature both in the paclitaxel- (Figure 3c) and the doxorubicin-resistant (Figure 3d) cells. Moreover, IGFBP3 was repressed in the latter ones (Figure 3d). The downregulation of IGFBP3 has been shown in non-small cell lung cancer (NSCLC) cell lines resistant to cisplatin [23] and EGFR tyrosine kinase inhibitors (TKIs) [24], as well as in advanced colorectal patients non-responsive to chemotherapy [25]. IGFBP5 expression was markedly decreased upon the acquisition of resistance to tamoxifen in MCF-7 breast cancer cells [26], and its suppression was suggested to be a mechanism of cisplatin resistance in esophageal squamous cell carcinoma [27]. Finally, IGFBP7 has also been associated with chemosensitivity, as a lower expression was noted in hepatocellular cancer cells resistant to interferon [28] and NSCLC cells resistant to cisplatin [29] compared to the parental cells.

To examine whether the protein products of the differentially expressed genes in chemoresistant cells could be functionally related, we plotted their network of interactions using data from the STRING database (Figure S2). In all cases, either for upregulated or downregulated genes, we found that the constructed networks had significantly more interactions than expected by chance (PPI enrichment *p*-value <1.0 × 10^−16^). There was a stronger network of interactions among the products of genes upregulated in the paclitaxel-resistant cells, which involved proteins such as VCAM1, NCAM1, CAV1, and FN1 (Figure S2a), which is indicative of the conversion of cells towards a more basal-like phenotype. In the case of upregulated genes in doxorubicin-resistant SUM159 cells, a strong network of interactions involving gene products related to cell cycle progression (CCNE1, CCNE2, CCNB1, CDC6, and CDK2, etc.) could be seen (Figure S2b). For downregulated genes in paclitaxel-resistant cells, there were strong connections among gene products related to an immune-inflammatory phenotype, such as CCL5, CCL3, IL1B, and IL7R (Figure S2c), which may indicate the loss of an epithelial-like phenotype (in accordance with [10]). An analogous inflammation-related network could be seen for downregulated gene products in doxorubicin-treated cells (Figure S2d).

**Figure 4:**
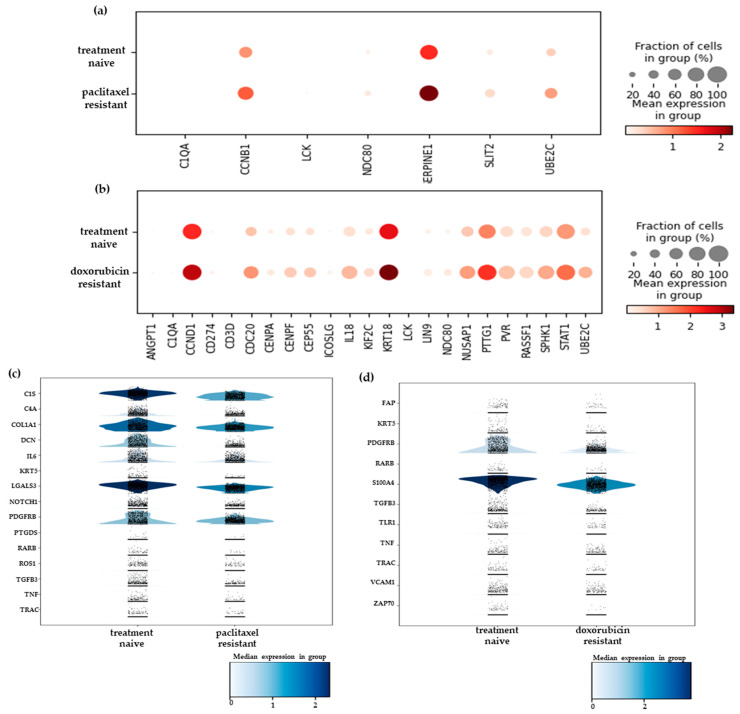
Differentially expressed genes identified by scRNA-seq only in the 3D- and not in the 2D-cultured chemoresistant SUM159 cells compared to the treatment-naïve ones. Dotplots depicting genes that are upregulated in the (**a**) paclitaxel- and(**b**) doxorubicin-resistant spheroids, respectively. Stacked violin plots depicting genes that are downregulated in the (**c**) paclitaxel- and (**d**) doxorubicin- resistant spheroids, respectively. Each dot represents the normalized gene expression per cell. The ZAP70 gene is missing from (**c**), and the PTGDS and IL17F genes are missing from (**d**) due to their very low counts in the chemoresistant cells that excludes them from the filtered count matrix.

We also extended this analysis to 3D spheroids, seeking to uncover the gene expression differences between the two in vitro culture systems. A pseudobulk analysis of counts of the scRNA-seq data generated from the 3D spheroids led to the identification of 22 and 60 upregulated genes in the paclitaxel- and doxorubicin- resistant cells, respectively, compared to the treatment-naïve spheroids (Tables S1 and S2). Most of these genes were also identified in the experiments with the 2D-grown cells (15/22 and 37/60). The genes uniquely upregulated in the spheroids and not in their monolayer counterparts are shown in Figure 4a,b. Among them, a gene commonly upregulated in both drug-resistant 3D models was *UBEC2C*, which encodes for ubiquitin-conjugating enzyme 2C. This gene was found overexpressed and has been associated with chemotherapy resistance in several tumor types, including breast (reviewed in [30]). In the paclitaxel-resistant spheroids, we also detected the overexpression of *SLIT2* (Figure 4a), a secreted glycoprotein that plays an important role in axon guidance and neuronal migration. It has not been well studied in cancer, but it was found to be upregulated in tamoxifen-resistant MCF-7 breast cancer cells [31]. Changes in sphingosine metabolism have been associated with drug resistance in several types of cancer. Our data showed an increase in the expression of a key enzyme, *SPHK1*, in the doxorubicin-resistant spheroids (Figure 4b), suggesting that some resistant cells may be targeted by inhibiting this enzyme. A downregulation of expression was displayed by 44 and 37 genes in the paclitaxel- and doxorubicin-resistant spheroids, respectively (Tables S3 and S4). When we examined the genes that were uniquely downregulated in the spheroid models, we identified 16 and 13 genes in the paclitaxel- (Figure 4c) and doxorubicin-resistant (Figure 4d) ones, respectively. Among these genes, the downregulation of *DCN* (Figure 4c) in the paclitaxel-resistant spheroids should be pointed out. This gene encodes for a proteoglycan that is a critical component of ECM, with important regulatory functions, and also exhibits a significant tumor suppressive effect [32,33]. Its role in therapy response has not been well studied, and the few studies conducted so far have yielded contradictory results [33]. Our data suggested that *DCN* may mediate sensitivity to paclitaxel in TNBC cells.

In conclusion, transcriptomic analysis at the single-cell level of TNBC chemoresistant cells revealed genes that may affect the response of TNBC cells to chemotherapeutics that had not been identified before due to their expression in scarce populations of resistant cells.

### 2.3. Heterogeneity in the Chemoresistant TNBC Populations Reflects Distinct Mechanisms of Resistance

**Figure 5:**
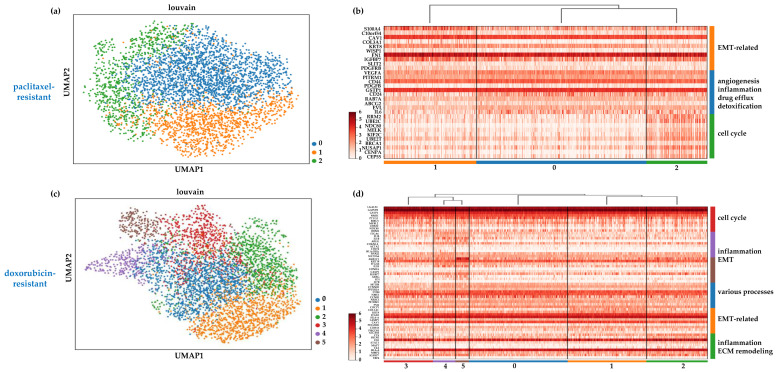
UMAP plots depicting clusters (subpopulations) of SUM159 (**a**) paclitaxel- and (**c**) doxorubicin-resistant cells grown in monolayer. The expression levels of the top 10 differentially expressed marker genes in each identified cluster are shown in heatmaps for the paclitaxel- (**b**) and the doxorubicin-resistant (**d**) cell states. Representative biological processes possibly mediating chemoresistance in each cell cluster are shown.

Our previous analysis (Section 2.1) indicated that the chemoresistant cell populations retained a degree of heterogeneity, implying that the various cell clusters comprising them may possess distinct mechanisms to evade cytotoxic drug effects. Aiming to pinpoint such mechanisms and reveal the characteristic therapeutic vulnerabilities for each cell group, we identified and visualized the top 10 differentially expressed genes in each one in the paclitaxel- and doxorubicin-resistant cells grown in a monolayer. These data are presented in Figure 5 and the top 100 differentially expressed genes are listed in Tables S5 and S6.

The results of this analysis confirmed that the paclitaxel-resistant cells in cluster 1 (depicted in orange in Figure 5a) resided in a highly mesenchymal state, since, besides the mesenchymal signature described above (Section 2.1), they also overexpressed genes known to induce EMT, including *S100A4* [34,35], *C10orf54* [36], and *WISP1* [37] (Figure 5b). Therefore, it is reasonable to assume that the EMT pathway is involved in the resistance phenotype of these cells and its targeting [38] may be a viable option to restore drug sensitivity. The epithelial-like cell cluster 2 (depicted in green in Figure 5a) was characterized by the upregulation of genes involved in cell cycle progression and mitosis, such as *RRM2*, *BE2C, NDC80, MELK, KIF2C,* and *CENPA* (Figure 5b). Furthermore, the presence of *BRCA1* suggests the rapid correction of DNA damage induced by doxorubicin. Targeting the cell cycle (for example, by using CDK inhibitors) and/or the DNA repair pathways could potentially contribute to the elimination of these cells. Cluster 0 cells (depicted in blue in Figure 5a) upregulated genes involved in several different biological processes that contribute to drug resistance and can, thus, be potential therapeutic targets (Figure 5b). *VEGFA* and *PDGFB* are angiogenic factors that could play roles in providing a supportive TME, therefore, anti-angiogenic drugs (e.g., bevacizumab for VEGFA) could be effective against this cell group. The interleukin 6 (IL-6) signaling pathway is crucial in mediating inflammation and has been implicated in cancer progression, metastasis, and drug resistance [39]. Several inhibitors of this pathway have been developed and clinical trials are currently underway to determine their effectiveness in various cancer types [39]. Such inhibitors may also be effective in targeting this subgroup of chemoresistant cells, where IL-6 is upregulated (Figure 5b). Finally, two genes that are widely overexpressed in drug-resistant cancers, the glutathione S-transferase Pi 1 (*GSTP1*) and *ABCG2*, a member of the ATP-binding cassette family, were also found in this cluster. *GSTP1* plays a crucial role in detoxifying chemotherapeutic drugs [40], and *ABCG2* mediates drug efflux [41]. Numerous inhibitors have already been developed for these two proteins and ongoing research aims to prove their clinical value, in combination or standalone therapy, in counteracting drug resistance [40,41].

In doxorubicin-resistant cells, the mesenchymal gene signature in cluster 1 (depicted in orange in Figure 5c) also suggested an activated EMT process that probably mediates increased drug resistance. This may be linked to the overexpression of *ITGB1* in this cluster (Figure 5d), a multi-functional cell adhesion molecule that is known to trigger EMT and promote drug resistance in cancer cells [42], suggesting that integrin signaling inhibitors could also disrupt mesenchymal features and sensitize cells to doxorubicin. The presence of the transcription factor *STAT1* (Signal transducer and activator of transcription 1) and the enzyme *MMP2* (matrix metallopeptidase 2) in cluster 2 (depicted in green in Figure 5c) implied roles for inflammation and extracellular matrix remodeling, respectively, in these cells, two processes that are interlinked and known to support cancer progression [43] and drug resistance [44]. Directly targeting MMPs or inhibiting their activation pathways could be a promising approach against these drug-resistant cells. It should be noted that the two most highly upregulated genes in this cluster were the complement serine protease *C1S* and the developmental transcription factor *EBF4* (Figure 5d). Very little is known about the roles of these genes in cancer, however, it is thought that the first one plays a role in suppressing anti-tumor immunity [45] and that the second one is part of the metastatic cascade in breast tumors [46]. Cluster 3 (depicted in red in Figure 5c) was dominated by the upregulation of cell-cycle-associated genes, similarly to cluster 2 in the paclitaxel-resistant cells, and could be targeted via the same means.

The epithelial-like clusters 4 and 5 (depicted in purple and brown, respectively, in Figure 5c) shared several upregulated genes, including the pro-inflammatory cytokine *IL1B*, which has been associated with chemoresistance [47]. Thus, its inhibition, for example, through the FDA-approved drug anakinra [48], could be beneficial. Furthermore, in cluster 5, the upregulation of the endothelin receptor A (*EDNRA*) gene may have enhanced the pleiotropic effects of its ligand, endothelin-1, which is known to regulate EMT, angiogenesis, and immune response and has been implicated in drug resistance in various tumor types [49]. An antagonist of EDNRA, macitentan, has been shown to sensitize colorectal cancer cells [50] and brain metastases [51] to chemotherapy, and it is plausible that it may help to overcome the resistance mechanisms in this TNBC cluster. Finally, cluster 0 (depicted in blue in Figure 5c) displayed the overexpression of genes (Figure 5d) that participate in various biological processes implicated in chemoresistance. The two most highly upregulated genes were *MTDH,* which encodes for the transmembrane protein metadherin that is involved in multiple aspects of cancer progression, metastasis, and drug resistance [52], and cyclin B1 (*CCNB1*), suggesting that cell cycle inhibitors may be effective against these cells.

Overall, the chemoresistant SUM159 cells comprised transcriptionally distinct subpopulations that upregulate genes involved in a wide array of cellular processes that may contribute to the development of chemoresistance, such as promoting EMT, facilitating DNA repair, and altering the cell cycle, etc. Our data suggest that targeting multiple pathways through combination therapies may be a more efficient approach to eliminate these cells and overcome chemoresistance in TNBC.

### 2.4. Rare Drug-Resistant Cells Preexist in the Treatment-Naïve Cell Populations

Next, we sought to investigate whether the high transcriptional variability at the single-cell level involved resistance-associated genes expressed highly but scarcely in the treatment-naïve cells. In our gene panel, two well-known markers of drug resistance were included, the ABC membrane transporters *ABCB1* and *ABCG2* [53]. The expression of both genes was strongly induced in the two chemoresistant cell states in the 2D amd in the 3D culture systems.

**Figure 6:**
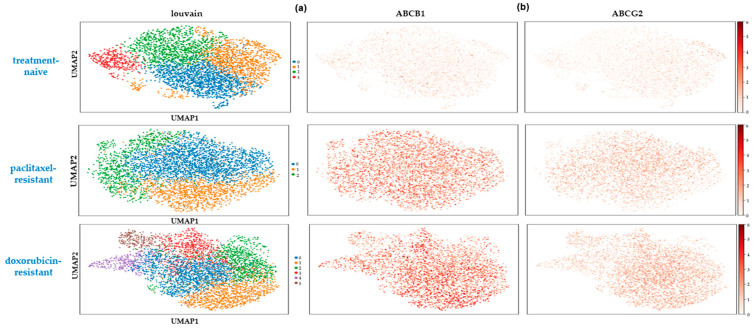
The expression patterns of the 2 ABC transporters *ABCB1* (**a**) and *ABCG2* (**b**) are shown in single cells of the different clusters of SUM159 treatment-naïve (**upper panel**), paclitaxel- (**middle panel**) and doxorubicin- (**lower panel**) resistant cells.

In the 2D-grown cells, the *ABCB1* gene was 30- and 50- fold upregulated in the paclitaxel- and doxorubicin- esistant cells respectively, showing an almost universal expression pattern (expressed in 92.6% of paclitaxel- and 87.4% of doxorubicin-resistant cells) (Figure 6a). The *ABCG2* gene was ~7-fold induced in paclitaxel- and ~10-fold induced in doxorubicin-resistant cells, being expressed in 80.7% and 87.3% of the respective populations (Figure 6b). Even though the robust upregulation of these two genes is a wide-spread mechanism of drug resistance in cancer, we found that they were both already expressed in the treatment-naïve cells, albeit in a very small percentage of them (Figure 6a,b). Specifically, 34.8% and 28.2% of the drug-naïve cells showed some expression of *ABCB1* and *ABCG2*, respectively, but only 0.8% and 0.7% of the cells had relatively high levels (>5 counts).

Similar results were obtained from the 3D spheroids. The *ABCB1* gene was 11-and 23-fold upregulated in the paclitaxel- and doxorubicin-resistant spheroids, respectively, showing a wide-spread expression pattern (expressed in 84.9% of paclitaxel- and 80.7% of doxorubicin-resistant cells). The *ABCG2* gene was ~3-fold overexpressed in both types of chemoresistant spheroids, with a more limited expression pattern (expressed in 57.9% of paclitaxel- and 56% of doxorubicin-resistant cells). In the treatment naïve-spheroids, a relatively high gene expression (>5 counts) was manifested by 3.4% and 2.3% of the cells for the *ABCB1* and *ABCG2* genes, respectively.

Collectively, these data suggest that rare, pre-defined cell states that are associated with chemoresistance exist before drug exposure. They may represent seeding subpopulations that possess a selective advantage over the bulk of tumor cells, enabling them to survive under drug pressure, develop additional mechanisms of acquired resistance, and regenerate the cell population.

## 3. Discussion

TNBC represents a highly heterogeneous and particularly aggressive subtype of breast cancer, usually combatted with conventional chemotherapeutic drugs, such as paclitaxel and doxorubicin. The efficacy of chemotherapy is often hampered by the innate and/or acquired chemoresistance that TNBC patients develop, leading to high rates of recurrence and fatal metastases [2,54]. The processes through which chemoresistant cells emerge remain somewhat ambiguous, yet they have been closely associated with intratumoral heterogeneity, which is particularly high in TNBC [4]. Therefore, bulk omics analyses may fail to dissect the distinct mechanisms of resistance developed by the different cell subpopulations that comprise a tumor, especially the minor ones.

Only a small number of studies have characterized the transcriptomic diversity of TNBC prior to and after the development of chemoresistance at the single-cell level. To fill in this gap, we employed scRNA-seq along with bulk RNA-seq to extensively analyze the transcriptomes of the TNBC cell line SUM159 and its chemoresistant derivatives grown as 2D or 3D cultures. Even though established cancer cell lines are presumed to be highly uniform, it has been recently confirmed that they exhibit some degree of heterogeneity that mimics patients’ intertumor transcriptomic diversity and drug responses [55].

Our experiments confirmed the presence of transcriptionally distinct subpopulations both in the parental and chemoresistant cells that resided in different states of the EMT spectrum, based on a gene signature generated from the intersection of published data [10] with our own. However, we noted a shift towards a highly mesenchymal phenotype in the drug-resistant cell lines, especially displayed in certain cell clusters. It is widely accepted that the EMT is a critical factor in tumor progression, metastasis, and drug resistance [38], even though the direct link between them remains a subject of debate. Notably, an EMT chemoresistance gene signature was identified in TNBC tumor samples after neoadjuvant chemotherapy, leading the authors to propose that targeting EMT signaling may resensitize cells to drugs [56]. Increasing evidence, however, indicates that multiple EMT-associated signaling pathways contribute to the emergence of drug-resistant cancer cells [38]. In our results, the upregulation of *PDGFRB* was a common element in the highly mesenchymal cell clusters in both drug-resistant cell lines, in accordance with the fact that PDGF signaling is mostly operational in mesenchymal cells [57]. Tyrosine kinase inhibitors that target PDGFRB are already in use for other malignancies [57] and could be applied in TNBC to combat EMT-driven resistance. Our scRNA-seq data also highlighted the FOXA1 transcription factor as a potential driver of resistance both to paclitaxel and doxorubicin. Even though *FOXA1* is considered to be a luminal gene, recent reports have shown that it is overexpressed in subsets of particularly aggressive TNBC tumors [17,18,19]. Our findings suggested that FOXA1 is upregulated in resistant TNBC cells, and future experiments should investigate whether its targeting improves cells’ sensitivity to chemotherapeutics. Even though it is notoriously hard to inhibit transcription factors, elucidation of the mechanisms of action of FOXA1 in TNBC may lead to the identification of druggable targets, as was recently shown in a subset of non-small cell lung cancer cell lines [58].

Additionally, our data revealed several differences in the gene expression profiles between cells grown in monolayer cultures and those in 3D spheroids. The 3D culture model exhibited unique resistance-associated gene expression patterns not observed in the 2D cultures. Taking into account the fact that 3D models are considered to be more physiologically relevant in cancer research, it may be advisable that they are more widely adopted in the study of tumor heterogeneity and chemoresistance at the single-cell level. Such an approach may uncover potential therapeutic targets that are overlooked in traditional 2D cultures.

An interesting observation in our data was the fact that chemoresistant subpopulations were not uniform, but they displayed considerable heterogeneity in their gene expression programs. The significance of this finding is evident; bulk transcriptomic analyses of cancer cells will identify the major mechanisms of resistance developed by dominant subclones or/and by most cells, but they will miss those employed only by minor groups of cells. The latter will have an advantage against therapies designed based on the prevailing mechanisms of chemoresistance. For example, the cells in cluster 5 of the doxorubicin-resistant population may not be affected by cell cycle inhibitors targeting clusters 0 and 3, but they may succumb to treatment with the repurposed drug macitentan that is approved to treat hypertension [59].

The presence of small numbers of primed cancer cells expressing resistant genes prior to drug exposure has been reported before in TNBC patients [56], as well as in MCF-7 luminal breast cancer cells [60]. In accordance with these data, we identified rare populations in the treatment-naïve cells expressing the multidrug efflux pumps *ABCB1* and *ABCG2*. The detection of such populations prior to the administration of chemotherapy in TNBC patients could predict the poor responders [56]. However, a whole transcriptome single-cell analysis of a large number of samples is required to characterize the pre-existing chemoresistant signatures in the different TNBC subtypes and determine their clinical value.

In conclusion, our findings highlight the critical role of scRNAseq in uncovering the rare cell populations and subtle gene expression changes that are often masked in bulk RNA-seq analyses. In TNBC, this translates to targeting multiple cellular subpopulations to effectively combat chemoresistance. Future therapeutic strategies could benefit from this approach by combining drugs that target both the dominant and minor resistant cell populations, potentially preventing tumor relapse and improving patient outcomes.

## 4. Materials and Methods

### 4.1. Cell Culture and Generation of Chemoresistant Cell Lines

The SUM159 TNBC cell line was a generous gift from Dr. R.W. Weinberg (Whitehead Institute, Boston, MA, USA) and was used for all experiments. The culturing of these cells and generation of the paclitaxel-resistant cell line were described before [8]. Similarly, the doxorubicin-resistant cells were generated by exposing them to escalating doses (0.05 μm–1 μM) of the drug for 2 days, followed by a recovery period in a drug-free medium.

### 4.2. Generation of 3D Spheroid Cultures

For the 3D spheroid cultures, SUM159 cells were embedded in Matrigel (Corning, New York, NY, USA) pre-coated 96-well plates at a density of 7500 cells per well and cultured in Ham’s F12 (LM-H1235/500, Biosera, Cholet, France), supplemented with 10% fetal bovine serum (Ref 10437-028, Gibco, ThermoFisher Scientific, Waltham, MA, USA). The spheroids were allowed to form and grow for 7–10 days before being subjected to further treatments and analysis.

### 4.3. RNA Extraction and Bulk-RNA Seq Experiments

Total RNA was extracted from the 2D cell cultures using the RNeasy Mini Kit (74104, Qiagen, Hilden, Germany) following the manufacturer’s instructions. The RNA concentration was assessed using the NanoDropTM 2000 (ThermoFisher Scientific, Waltham, MA, USA). Libraries were prepared using the The NEBNext Ultra II Directional RNA Library Prep (E7760, Ipswich, MA, USA) according to the manufacturer’s instructions. The quality and quantity of the generated libraries were checked using the Bioanalyzer (Agilent, Santa Clara, CA, USA) and high-quality ones were sequenced in a 100-bp single-end mode on an Illumina NovaSeq 6000 platform (Illumina, San Diego, CA, USA) at the Greek Genome Center located at BRFAA.

### 4.4. Sample Preparation and scRNA-Seq Experiments

The preparation and evaluation of cells before the scRNA-seq experiments were performed using the BD Rhapsody™ Single-Cell Analysis System (BD Biosciences, Franklin Lakes, NJ, USA), following the manufacturer’s protocol. Briefly, single cell suspensions from the 2D and 3D cultures were labeled, and cell viability and number were evaluated using the BD Rhapsody™ scanner. Cell samples with a high viability (>85%) were used for sequencing. Cells grown in a monolayer or in 3D spheroids were pooled together and loaded onto two BD Rhapsody cartridges. The BD Rhapsody™ Onco-BC Panel Hs (633752, BD, Biosciences) was used for library construction according to the manufacturer’s instructions. The quality and quantity of the generated libraries were checked using the Bioanalyzer (Agilent, Santa Clara, CA, USA), and high-quality ones were sequenced in a paired-end mode on a NextSeq1000 Sequencer (Illumina, San Diego, CA, USA).

### 4.5. Data Analysis

#### 4.5.1. Bulk RNA-Seq Analysis

Bioinformatics analyses were performed within the Galaxy suite environment [61]. The quality of the sequencing reads was assessed with the FastQC algorithm. For the bulk RNA-seq analysis, reads were mapped to the hg19 version of the human genomec vx using HISAT2 [62]. The reads inside genes were calculated with HTSeq count [63]. A differential gene expression analysis was performed with DESeq2 [64]. Differentially expressed genes between the naïve and chemoresistant states were considered as those having a log2 fold-change > 1, padj < 0.05 and a minimum mean count of 20 reads. Raw RNA-seq data for the SUM159 attached treatment-naïve and paclitaxel-resistant cells were retrieved from GEO accession GSE206242 [8]. Raw RNA-seq data for SUM159 ITGB4-hi and p63/p74 double knockout cells were retrieved from GSE172407 [10].

#### 4.5.2. Targeted Single-Cell RNA-Seq Analysis

FASTQ files obtained from sequencing were provided to the Seven Bridges Genomics platform for further analyses. The quality-filtered R1 reads were analyzed to identify the cell label sequence (CL), Molecular Identifier sequence (UMI), and poly-dT tail sequence. Then putative cell information was combined together with RSEC-adjusted molecules to generate a single-cell expression matrix. Count matrices were then analyzed using the Scanpy package [9] from within the Galaxy suite (https://galaxy.inf.ethz.ch/) [61]. Counts were filtered to remove genes with extremely low counts and cells with low counts using the ‘Filter with Scanpy option’. The counts were normalized, logarithmized, and filtered for highly variable genes with Scanpy. The effect of cell cycle on cluster identification was ameliorated using the remove confounders option of Scanpy. Dimensionality reduction with PCA was followed by the computation of a neighborhood graph and clustering using the Louvain algorithm to identify clusters. The top 100 marker genes for each cluster were calculated with a t-test statistic. UMAP plots, heatmaps, and dotplots were produced with the ‘plot with Scanpy option’. To identify differentially expressed genes, the counts for each gene within each condition were aggregated and compared between conditions. Differentially expressed genes between conditions were considered as those having at least 2-fold difference in counts and a minimum count of 50 reads in the resistant state.

#### 4.5.3. STRING Analysis

To identify protein–protein interactions among the protein products of genes differentially expressed between treatment-naïve and chemoresistant cells, the gene lists were given as input to the STRING tool [65] and plotted by using the default parameters.

## Data Availability

Bulk and single cell RNA-seq have been deposited at GEO under accession GSE266356. To review the data please enter the token provided in the cover letter.

## Supplementary Materials

The supporting information can be downloaded at: https://www.mdpi.com/article/10.3390/ijms25136853/s1.
