# Supplementary material for: Identification of New Chemoresistance-Associated Genes in Triple-Negative Breast Cancer by Single-Cell Transcriptomic Analysis
Source: Int J Mol Sci. 2024 Jun 22;25(13):6853. doi: 10.3390/ijms25136853 (PMC11241600; doi:10.3390/ijms25136853)
Supplement: Supplementary file 1 [file ijms-25-06853-s001.zip › Supplementary Materials.pdf]

Supplementary Materials

Supplementary Figure S1. Differentially expressed genes in the 2D-cultured chemoresistant SUM159 cells compared to the treatment-naïve ones that were identified both by scRNA- and bulk RNA-seq. Dotplots depicting genes that are upregulated in the (a) paclitaxel- and(b) doxorubicin- resistant cells, respectively. Stacked violin plots depicting genes that are downregulated in the (c) paclitaxel- and (d) doxorubicin- resistant cells, respectively. Each dot represents the normalized gene expression per cell.

Supplementary Figure S2. Protein-protein interaction networks in chemoresistant SUM159 cells depicted using STRING. (a) Genes upregulated in paclitaxel-resistant SUM159 cells grown in 2D. (b) Genes upregulated in doxorubicin-resistant SUM159 cells grown in 2D. (c) Genes downregulated in paclitaxel-resistant SUM159 cells grown in 2D. (D) Genes downregulated in doxorubicin-resistant SUM159 cells grown in 2D.

Table S1. Genes upregulated in 3D paclitaxel-resistant spheroids identified by scRNA-seq.
Table S2. Genes upregulated in 3D doxorubicn-resistant spheroids identified by scRNA-seq.
Table S3. Genes downregulated in 3D paclitaxel-resistant spheroids identified by scRNA-seq.
Table S4. Genes downregulated in 3D doxorubicn-resistant spheroids identified by scRNA-seq.
Table S5. The top 100 genes differentially expressed in each cluster in the paclitaxel-resistant cells grown in monolayer.
Table S6. The top 100 genes differentially expressed in each cluster in the doxorubicin resistant cells grown in monolayer.

Figure S1.

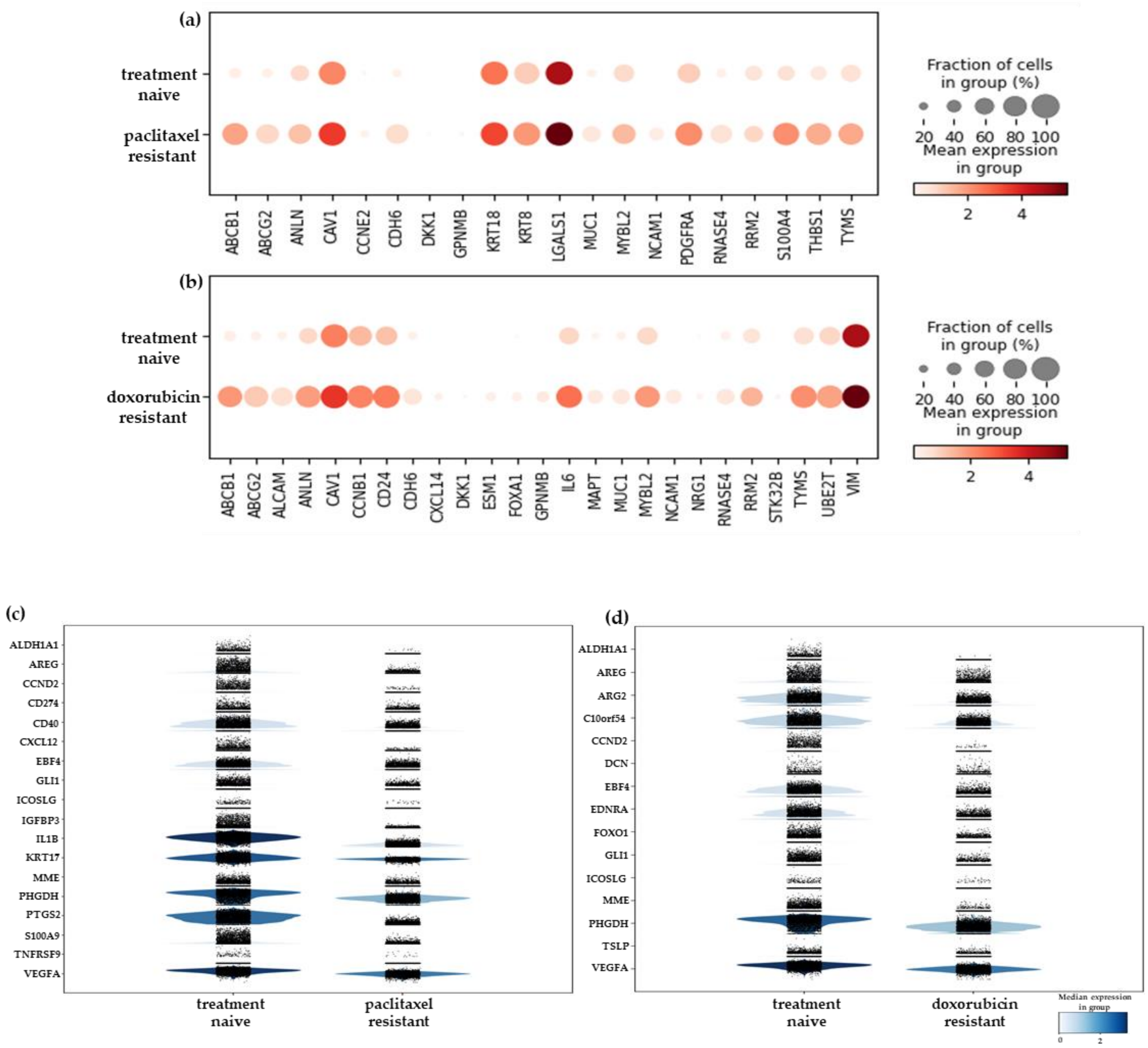
(a)
treatment naive
paclitaxel resistant
ABCB1
ABCG2
ANLN
CAV1
CCNE2
CDH6
DKK1
GPNMB
KRT18
KRT8
LGALS1
MUC1
MYBL2
NCAM1
PDGFRA
RNASE4
RRM2
S100A4
THBS1
TYMS
Fraction of cells in group (%)
20 40 60 80 100
Mean expression in group
2 4
(b)
treatment naive
doxorubicin resistant
ABCB1
ABCG2
ALCAM
ANLN
CAV1
CCNB1
CD24
CDH6
CXCL14
DKK1
ESM1
FOXA1
GPNMB
IL6
MAPT
MUC1
MYBL2
NCAM1
NRG1
RNASE4
RRM2
STK32B
TYMS
UBE2T
VIM
Fraction of cells in group (%)
20 40 60 80 100
Mean expression in group
2 4
(c)
ALDH1A1
AREG
CCND2
CD274
CD40
CXCL12
EBF4
GLI1
ICOSLG
IGFBP3
IL1B
KRT17
MME
PHGDH
PTGS2
S100A9
TNFRSF9
VEGFA
treatment naive
paclitaxel resistant
(d)
ALDH1A1
AREG
ARG2
C10orf54
CCND2
DCN
EBF4
EDNRA
FOXO1
GLI1
ICOSLG
MME
PHGDH
TSLP
VEGFA
treatment naive
doxorubicin resistant
Median expression in group
0 2

Tables

Table S1-S6. See the excel file entitled **"Suppl. Tables"**
