# Supplementary figures and images for: Identification of New Chemoresistance-Associated Genes in Triple-Negative Breast Cancer by Single-Cell Transcriptomic Analysis
Source: Int J Mol Sci. 2024 Jun 22;25(13):6853. doi: 10.3390/ijms25136853 (PMC11241600; doi:10.3390/ijms25136853)

(a)

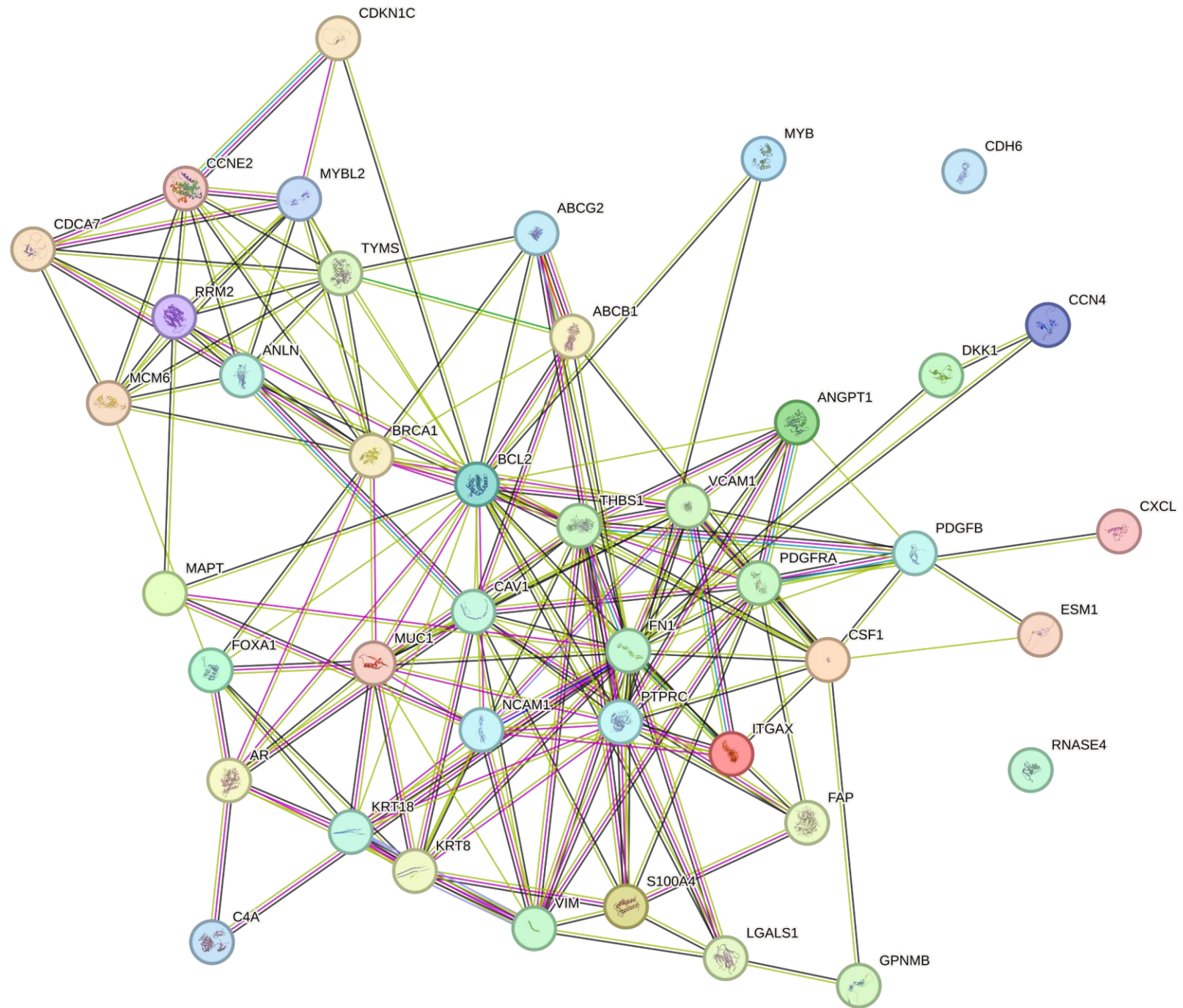

(b)

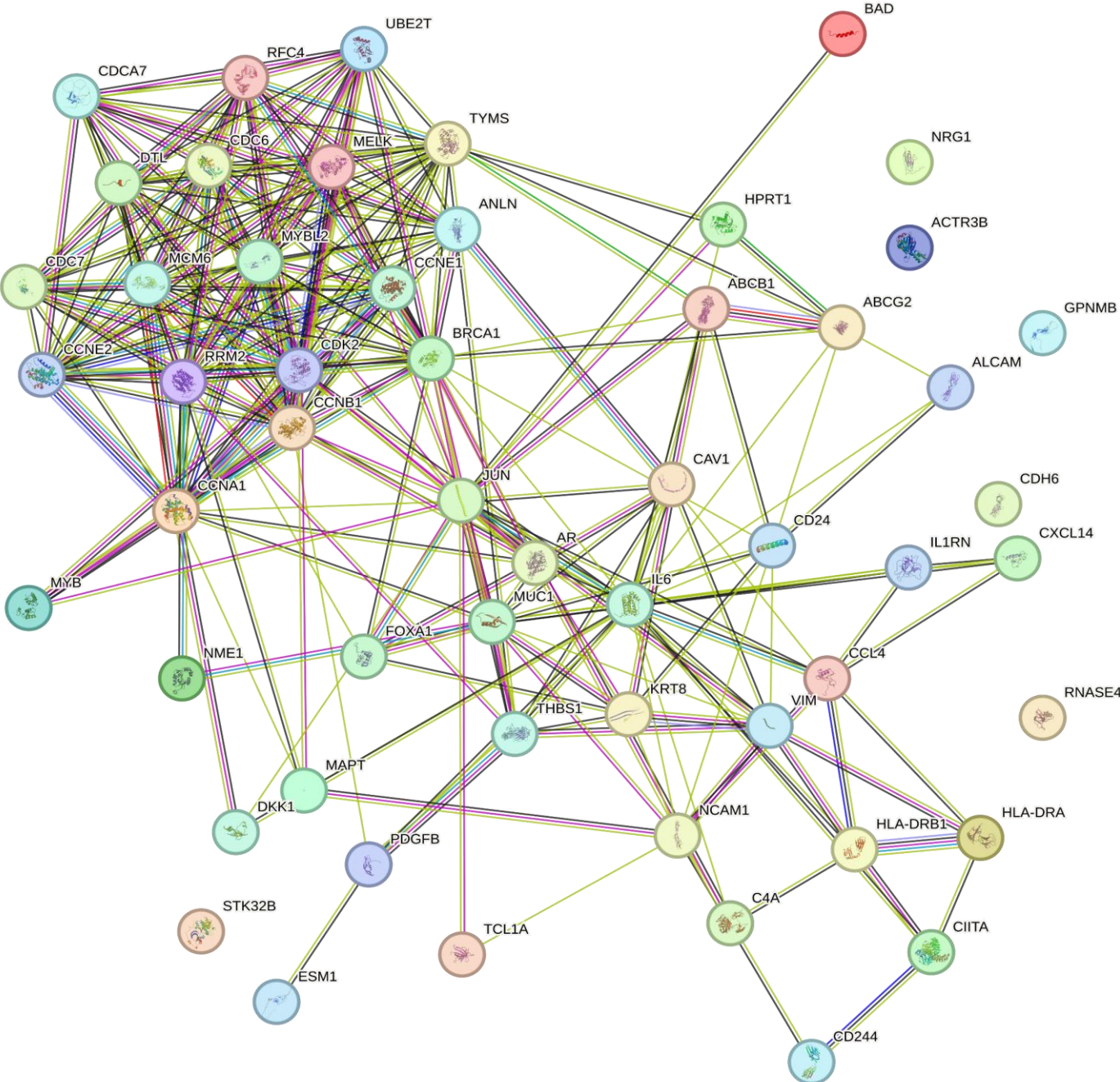

(c)

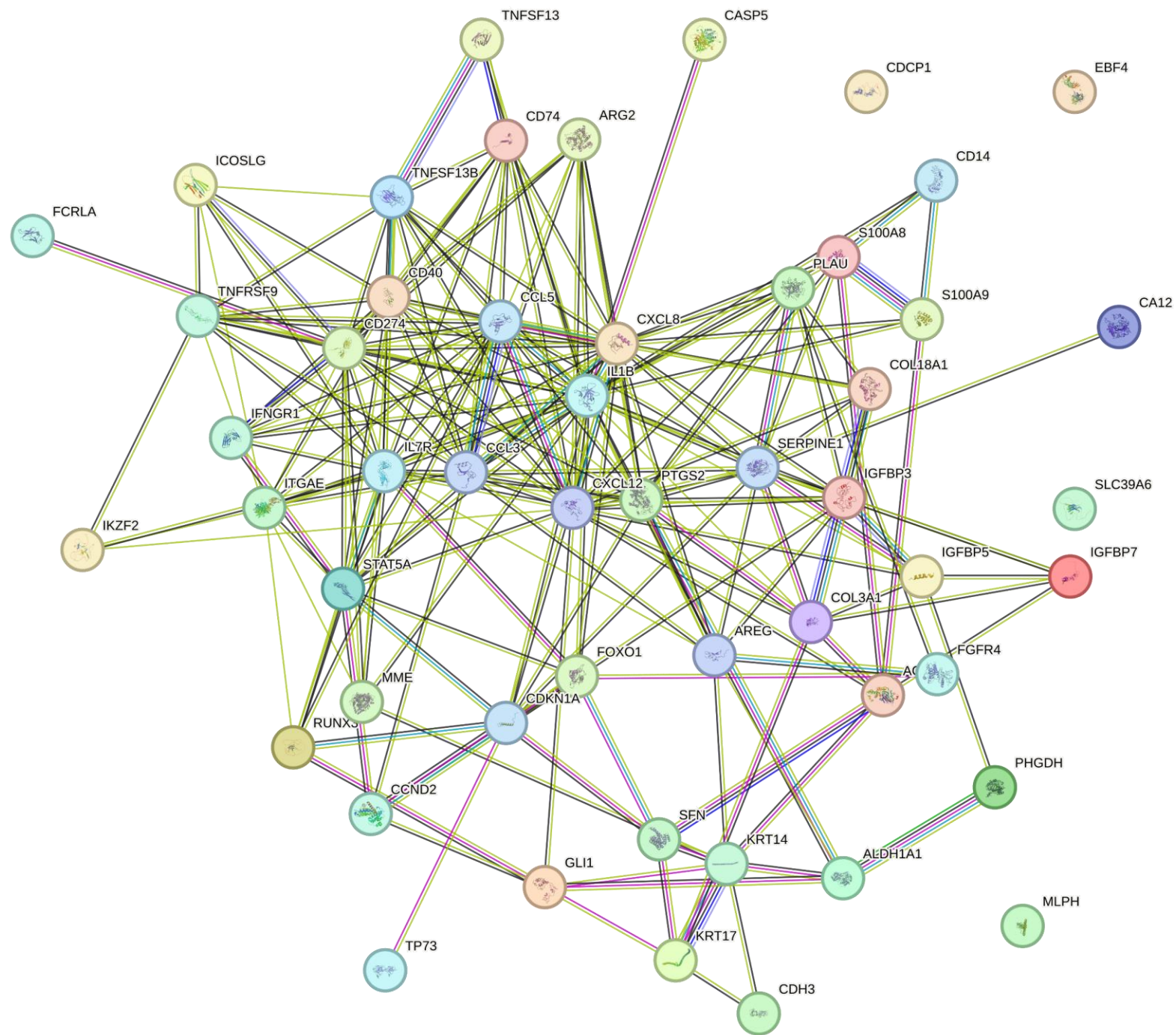

(d)

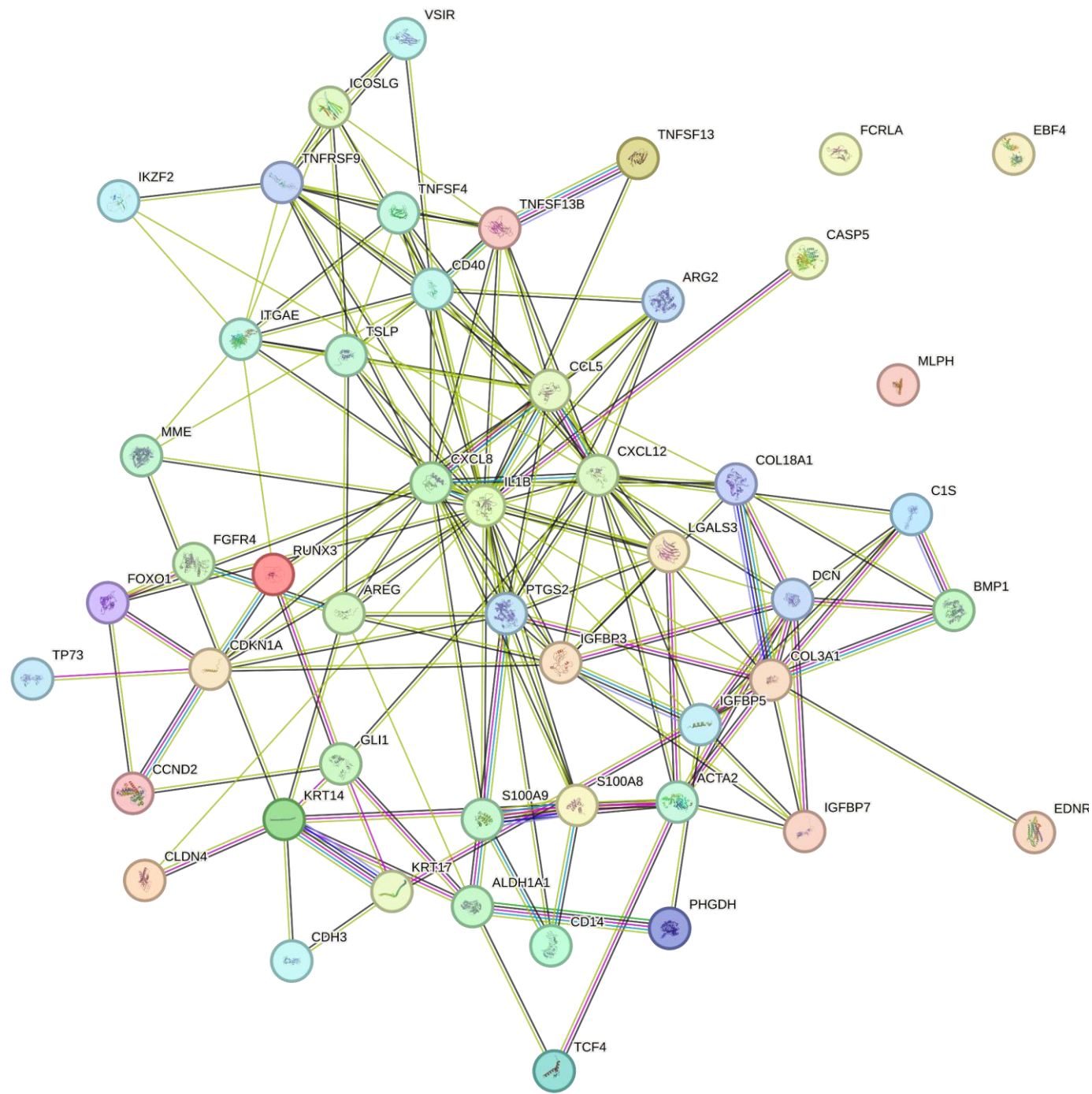

Supplement: Supplementary file 1 [file ijms-25-06853-s001.zip › Supplementary Figure S2.pdf]
